# Whipple’s Disease: A Case Report

**DOI:** 10.7759/cureus.39963

**Published:** 2023-06-05

**Authors:** Marta Batista, Maria Luis Santos, Cristina Silva, Gabriela Pereira, Glória Alves, Jorge Cotter

**Affiliations:** 1 Internal Medicine, Hospital da Senhora da Oliveira, Guimarães, PRT

**Keywords:** whipple’s disease, skin pigmentation, unintentional weight loss, persistent diarrhea, chronic abdominal pain

## Abstract

Whipple’s disease (WD) is caused by *Tropheryma whipplei*, frequently found in lamina propria’s macrophages in the small intestine. It is a rare and chronic systemic infection, and the principal clinical manifestations are diarrhea, weight loss, abdominal pain, and arthralgia. The diagnosis is difficult mainly because of its rarity and should be considered in patients with arthralgias, diarrhea, abdominal pain, and weight loss after more common conditions have been excluded. The laboratory diagnosis is established by a duodenal biopsy. The treatment involves 14 days of intravenous antibiotics with good penetration in the cerebrospinal fluid (i.e., ceftriaxone) and one-year treatment with oral co-trimoxazole. Early diagnosis and proper treatment are crucial because it improves the prognosis.

We report the case of a 58-year-old female with skin hyperpigmentation, loss of appetite and weight (16% of body weight in three months), nausea, upper abdominal pain, and diarrhea. Esophagogastroduodenoscopy and colonoscopy were performed to obtain biopsy samples, which, together with laboratory tests and microbiological studies, led to a diagnosis of Whipple’s disease.

## Introduction

Whipple’s disease (WD) is a rare chronic systemic infectious disease caused by *Tropheryma whipplei*, which is present in environmental water and soil and affects those who contact with contaminated water. It is found in the macrophages of the lamina propria of the small intestine, and its main manifestations are weight loss due to malabsorption, diarrhea, and abdominal pain [1-4]. WD additionally infects the nervous system, heart, and skin [3]. Possibly, there is an immunological defect in the T helper cell type 1 (Th1) cytokine pathway, such as interferon-γ or interleukin-12, that could explain the infection by *Tropheryma whipplei* [2,5]. The bacterium depends on and uses metabolic pathways from macrophages in the small intestine. The wall of the microorganism is made of mucopolysaccharides, which are stained red by the periodic acid-Schiff (PAS) reaction [3,6].

The diagnosis is based on esophagogastroduodenoscopy and duodenal biopsies that show foamy macrophages that are PAS-positive in the lamina propria, and the intestinal columnar is atrophic. If other organs are involved, such as the liver, lymph nodes, heart, central nervous system, or synovial membrane and WD is suspected, a biopsy should be done [2,3,7,8]. Nowadays, it is possible to use other tools, such as polymerase chain reaction (PCR) tissue analysis, which uses oligonucleotide probes to target repeated sequences of the microorganism and detect its DNA [2]. It is difficult to diagnose mainly because of its rarity and should be considered in patients with the cardinal manifestations (arthralgias, diarrhea, abdominal pain, and weight loss) after more common conditions have been excluded (inflammatory bowel disease, infectious causes of chronic diarrhea, connective tissue disease, advanced HIV infection, tuberculosis, and hyperthyroidism) [9].

The treatment is controversial and involves two weeks of intravenous antibiotics with good penetration in the blood-brain barrier (i.e., ceftriaxone) and one-year treatment with oral trimethoprim-sulfamethoxazole twice a day [2,10]. Sometimes, the microorganism persists for a long time in the tissues, and there are relapses with clinical deterioration, even with prolonged antibiotic regimens [2].

## Case presentation

A 58-year-old female presented to the emergency department with progressive weight loss, abdominal pain, nausea, and diarrhea. Over the past three months, she had been experiencing skin hyperpigmentation, loss of appetite and weight, nausea, abdominal pain, and diarrhea without triggering, aggravating, or relieving factors. The patient had a weight loss of 8 kg (16% of body weight) during the previous three months, and 3 kg in the last week. She denied smoking, alcohol, and drug consumption. She had no surgical history or previous hospitalizations and no drug allergies. She denied risky sexual contacts, recent travels, and contacts with animals.

The patient was hemodynamically stable, afebrile, conscious, and oriented, with no neurological focus on examination. Physical examination showed an emaciated female with skin hyperpigmentation and pain on deep abdominal palpation in the epigastrium and hypogastric quadrants. The patient did not have palpable nodules or joint changes, nor was there any alteration on cardiac or pulmonary auscultation.

Her laboratory findings are presented in Table 1 and showed hypochromic microcytic anemia and hypoalbuminemia. Her C-reactive protein, lactic dehydrogenase, and calprotectin were elevated.

**Table 1:** Laboratory investigations INR: international normalized ratio

Analyte	Patient value			Reference range
	On presentation	Discharge	One year	
Hemoglobin (g/dL)	11.2	11.1	12.5	12-16
Hematocrit (%)	35.3	35.3	37.7	40-50
Mean corpuscular volume (fL)	77.4	79.5	89.5	83-103
Mean corpuscular hemoglobin (pg)	24.6	25	29.7	28-34
Mean corpuscular hemoglobin concentration (g/dL)	31.7	31.4	33.2	32-36
Leukocytes (×10^3^/µL)	6.7	8.9	6.1	4.8-10.8
Neutrophils (%)	76.2	67.2	32.2	38-70
Lymphocytes (%)	14.4	23.8	60.4	20-40
Basophils (%)	0.6	0.6	0	0-1
Eosinophils (%)	4.2	2.7	2	1-6
Monocytes (%)	4.1	5.4	5.1	2-10
Platelets (×10^3^/µL)	303	416	153	150-300
C-reactive protein (mg/L)	17.8	11.9	0.8	<3
Total bilirubin (mg/dL)	<0.2	<0.20	0.51	0.3-1.2
Aspartate aminotransferase (IU/L)	38	30	42	12-40
Alanine aminotransferase (IU/L)	30	29	47	7-40
Gamma-glutamyl transferase (IU/L)	18	35	19	0-73
Lactic dehydrogenase (IU/L)	280	176	194	120-246
Alkaline phosphatase (IU/L)	106	100	62	46-116
Albumin (g/dL)	2.4	3.4	4.6	3.4-5
Calprotectin (µg/g)	519	-	-	<50
INR	1	1	1	

Connective tissue disease, advanced HIV infection, tuberculosis, and hyperthyroidism were excluded. The serologic evaluation to detect celiac disease was made and was negative. Microbiological and parasitological studies of the feces were carried out, and no agent was isolated. An upper gastrointestinal endoscopy showed erythematous gastropathy, and gastric biopsies revealed *Helicobacter pylori *(*H. pylori*) infection. In the colonoscopy, an erythematous ileal mucosa was visualized, and the duodenal and ileal biopsies performed revealed lamina propria with foamy macrophages that are PAS-positive, compatible with Whipple’s disease. The DNA from *T. whipplei *was detected in the blood by a polymerase chain reaction (PCR) assay. Those findings led to the diagnosis of the disease.

*Helicobacter pylori *eradication therapy and targeted therapy for Whipple’s disease with 14 days of ceftriaxone (2 g intravenous (IV) once daily) and co-trimoxazole (one double-strength tablet (160 mg trimethoprim/800 mg sulfamethoxazole) twice a day) for one year were prescribed. After the completion of antibiotic therapy, there was a substantial improvement in the patient’s clinical condition, with weight gain and no reappearance of previous symptoms. At this time, the patient repeated endoscopic studies without visible alterations in the duodenal and ileal mucosa but histologically maintained the PAS-positive macrophage infiltration in the lamina propria, although to a lesser degree.

## Discussion

It is important to study the differential diagnoses that occur in patients with diarrhea and weight loss of prolonged evolution, and although Whipple’s disease is rare, it becomes a diagnostic hypothesis. Some cases could be fatal if left untreated [2,11].

Our case report describes a patient who had three classical manifestations (weight loss, diarrhea, and abdominal pain), with skin hyperpigmentation, and she was diagnosed with Whipple’s disease during the study to determine the etiology of the clinical condition. In this case, there was no involvement of other organs, but there are cases in which there is, and that allows us to think about the diagnosis more urgently, namely, when there are manifestations such as seizures, gait changes, dementia, psychiatric disorders, arthralgias or arthritis, or ocular, pulmonary, cardiac, or hematologic involvement [2,12,13].

Possible conditions of gastrointestinal infection, hyperthyroidism, connective tissue disease, advanced HIV infection, and tuberculosis were excluded. Given the clinical manifestations and age of the patient, other more frequent hypotheses to be studied would be inflammatory bowel disease or celiac disease, although skin hyperpigmentation is rare in celiac disease and, in the case of inflammatory bowel disease, there are associated skin manifestations to immunological mechanisms, vitamin deficits, or adverse drug effects. As previously described, the serologic evaluation to detect celiac disease was made and was negative. Endoscopic evaluation of our patient showed an erythematous ileal mucosa. Histological examination of duodenal and ileal biopsies and PCR blood analysis revealed and confirmed the diagnosis of Whipple’s disease. Therefore, it is important not to devalue rare causes that could justify the patient’s clinical condition.

In this case, the treatment used was one of those referred to in the scientific literature that is associated with a good prognosis, lesser relapses, and consisted of 14 days with ceftriaxone and then one year of continuous treatment with oral antibiotics with co-trimoxazole twice a day [2,8-10]. At this time, the patient was reevaluated, with full recovery of the lost weight, without previous clinical manifestations, and repeated endoscopic studies without visible alterations in the duodenal and ileal mucosa but histologically maintained the PAS-positive macrophage infiltration in the lamina propria, although to a lesser degree, which shows a good prognosis. These histological findings could last for years, and if there is no clinical deterioration, the disease is not considered active [8,14]. Therefore, it is important to follow up with the patient to assess the clinical evolution and possible relapses.

## Conclusions

Whipple’s disease is a rare infectious multisystemic disease that is challenging to diagnose, and it should be considered in patients with typical manifestations such as arthralgias, diarrhea, abdominal pain, and weight loss after more common conditions have been excluded. It can occur in fatal courses, particularly in patients with cerebral or severe gastrointestinal involvement with malnutrition and electrolyte disturbances. If the diagnosis is timely, there is effective therapy with resolution of the clinical picture. It is important to follow up and monitor for relapses that can occur despite complete antibiotic treatment.
